# Toll-Like Receptors: Expression and Roles in Otitis Media

**DOI:** 10.3390/ijms22157868

**Published:** 2021-07-23

**Authors:** Su Young Jung, Dokyoung Kim, Dong Choon Park, Sung Soo Kim, Tong In Oh, Dae Woong Kang, Sang Hoon Kim, Seung Geun Yeo

**Affiliations:** 1 Department of Otorhinolaryngology—Head and Neck Surgery, Myongji Hospital, Hanyang University College of Medicine, Goyang 10475, Korea; monkiwh35@hanmail.net; 2 Department of Anatomy and Neurobiology, College of Medicine, Kyung Hee University, Seoul 02447, Korea; dkim@khu.ac.kr; 3 Department of Obstetrics and Gynecology, St. Vincent’s Hospital, College of Medicine, The Catholic University of Korea, Suwon 16247, Korea; park.dongchoon@gmail.com; 4 Department of Biomedical Engineering, College of Medicine, Kyung Hee University, Seoul 02447, Korea; sgskim@khu.ac.kr; 5 Department of Biochemistry and Molecular Biology, School of Medicine, Kyung Hee University, Seoul 02447, Korea; tioh@khu.ac.kr; 6 Department of Otorhinolaryngology—Head and Neck Surgery, School of Medicine, Kyung Hee University, 23, Kyung Hee Dae-ro, Dongdaemun-gu, Seoul 130872, Korea; kkang814@naver.com (D.W.K.); hoon0700@naver.com (S.H.K.)

**Keywords:** Toll-like receptor, otitis media, acute otitis media, otitis media with effusion, chronic otitis media, chronic otitis media with cholesteatoma

## Abstract

Otitis media is mainly caused by upper respiratory tract infection and eustachian tube dysfunction. If external upper respiratory tract infection is not detected early in the middle ear, or an appropriate immune response does not occur, otitis media can become a chronic state or complications may occur. Therefore, given the important role of Toll-like receptors (TLRs) in the early response to external antigens, we surveyed the role of TLRs in otitis media. To summarize the role of TLR in otitis media, we reviewed articles on the expression of TLRs in acute otitis media (AOM), otitis media with effusion (OME), chronic otitis media (COM) with cholesteatoma, and COM without cholesteatoma. Many studies showed that TLRs 1–10 are expressed in AOM, OME, COM with cholesteatoma, and COM without cholesteatoma. TLR expression in the normal middle ear mucosa is absent or weak, but is increased in inflammatory fluid of AOM, effusion of OME, and granulation tissue and cholesteatoma of COM. In addition, TLRs show increased or decreased expression depending on the presence or absence of bacteria, recurrence of disease, tissue type, and repeated surgery. In conclusion, expression of TLRs is associated with otitis media. Inappropriate TLR expression, or delayed or absent induction, are associated with the occurrence, recurrence, chronicization, and complications of otitis media. Therefore, TLRs are very important in otitis media and closely related to its etiology.

## 1. Introduction

### 1.1. Overview of Otitis Media

Otitis media, a group of inflammatory diseases of the middle ear, are among the most common diseases in infants and children. In particular, recurrent or chronic otitis media (COM) in children cause hearing loss, which can lead to speech development disorders, delayed language acquisition, attention deficit disorders, and behavioral abnormalities in children. Most acute otitis media (AOM) cases improve without complication, but often transition to recurrent or persistent otitis media or otitis media with effusion (OME) and COM if inflammation in the middle ear cavity is not effectively treated [1,2]. The causes of otitis media are diverse and include interactions between various factors that affect the middle ear cavity. However, what causes acute infections in the middle ear and mastoid cavity to develop into chronic inflammation is incompletely understood.

Otitis media can be classified according to its associated clinical symptoms, otoscopic findings, duration, frequency, pathology, and complications into AOM, OME (residual or persistent effusion), and COM with or without cholesteatoma. AOM and OME are inflammatory conditions that are histologically detectable, mainly in the inner and outer fibrous layers of the lamina propria, and they affect the elasticity of the tympanic membrane, causing its retraction or perforation [3,4]. In COM, various histopathological changes occur in the middle ear and mastoid cavity. Some appear as a direct result of infection and others as a result of the host’s immune response. COM without cholesteatoma causes changes in the mucosa, submucosa and surrounding bony tissue as a result of a persistent inflammatory reaction in the middle ear cavity or mastoid cavity. Important pathological findings of COM include granulation tissue formation, bone changes, tympanosclerosis, cholesterol granuloma, cholesteatoma and fibrosis, all of which are irreversible [5,6]. In COM with cholesteatoma, keratinized squamous epithelium invades into the mucosal membrane of the middle ear cavity, causing accumulation of keratin and destruction of surrounding bony tissue. In particular, cholesteatoma or granulation tissue in COM can cause bone destruction, clinically manifesting as hearing loss, dizziness, facial nerve palsy, and intracranial complications [7,8].

### 1.2. Toll-Like Receptors as Pattern Recognition Receptors (PRRs)

The main initial response to pathogenic microorganisms introduced into the human body is an innate immune response in which recognition of pathogen-associated molecular patterns (PAMPs) or microbe-associated molecular patterns (MAMPs) triggers intracellular signals that produce various cytokines and chemokines. Accordingly, the innate immune response serves as a primary defense mechanism or induces an acquired immune response. If the pathogen is not recognized early and accurately, chronic or recurrent disease as well as permanent, fatal damage may occur in the host. Therefore, early recognition of pathogens is a very important aspect of host defense mechanisms. Host cell pattern-recognition receptors (PRRs), which bind PAMPs or MAMPs on pathogens, are important in this early recognition. In innate immunity, some PRRs recognize PAMPs that are characteristic of microbial targets. The fact that a limited variety of PRRs recognizes diverse pathogens is actually a very important aspect of our immune system. PRRs are classified according to their ligand specificity, function, localization and/or evolutionary relationships. Based on their localization, PRRs may be divided into membrane-bound PRRs and cytoplasmic PRRs. Membrane-bound PRRs include Toll-like receptors (TLRs) and C-type lectin receptors (CLRs). Cytoplasmic PRRs include NOD-like receptors (NLRs) and RIG-I-like receptors (RLRs) [9,10]. 

TLRs are a class of proteins that play key roles in the innate immune system. There are 13 different TLRs, TLR1–13, of which all but TLR11–13 are expressed in humans [11,12]. TLRs recognize a set of molecular modalities that are not normally found in vertebrates, allowing them to differentiate between self and pathogens and thus play important roles in the host’s primary defense. TLRs are found in immune cells such as dendritic cells, macrophages, neutrophils, T cells and B cells as well as in non-immune cells such as fibroblasts, epithelial cells, and melanocytes. These TLRs serve as the primary defense against infection [13]. TLRs share a leucine-rich extracellular domain preceded by a cysteine-rich site. The structure of the cytoplasmic domain, called the Toll interleukin-1 receptor (TIR) domain, is similar to that of the interleukin-1 (IL-1) receptor. Signal transduction by TLRs converges on activation of nuclear factor kappaB (NF-кB); this process is mediated by the TIR domain, which interacts with the TIR domain-containing ligand, MyD88 (myeloid differentiation primary response gene 88), to form a complex [14]. In addition to forming a complex with the TLR via its C-terminal TIR domain, MyD88 forms a complex with Ser/Thr kinases IRAK1 (interleukin 1 receptor-associated kinase 1) or IRAK2 via its N-terminal death domain. IRAK1, in turn, acts through interactions with TRAF6 (TNF receptor-associated factor 6) to activate NIK (NF-кB–inducing kinase), which phosphorylates IκB kinase (IKK). Phosphorylated IKK then phosphorylates the NF-κB inhibitor, IκB, thereby activating NF-κB. Finally, activated NF-κB moves to the nucleus, where it acts as a transcription factor to induce expression of cytokines and other bioactive molecules, serving as a bridge between innate and adaptive immune responses [15,16].

**Figure 1:**
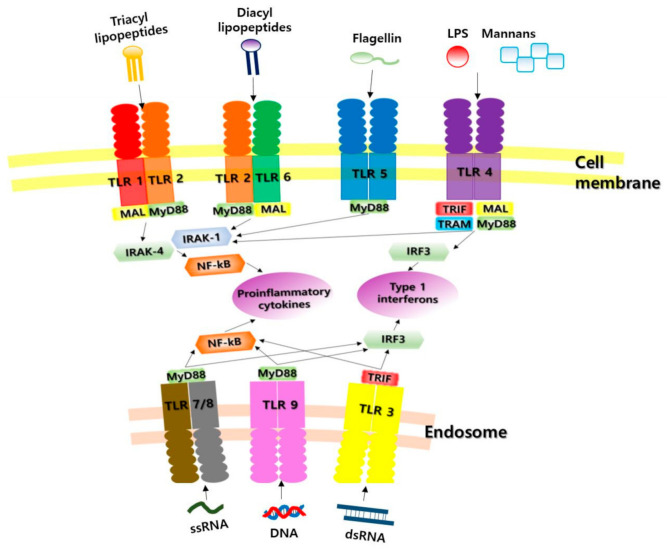
Ten major classes of Toll-like receptors and their most important ligands. TLR, Toll-like receptor; LPS, lipopolysaccharide; MyD88, myeloid differentiation factor 88; MAL, myelin and lymphocyte protein; TRIF, Toll-like-receptor adaptor molecule; TRAM, TIRF-related adaptor molecule; IRF3, interferon regulatory factor 3; NF-kB, nuclear factor-kB; IRAK-1, nterleukine-1 receptor-associated kinase 1; IRAK-4, nterleukine-1 receptor-associated kinase 4.

TLRs detect bacteria, viruses, fungi and protozoa, triggering an innate immune response as described above. Microbial products capable of stimulating TLR signals include lipopolysaccharides (LPS) from Gram-negative bacteria, peptidoglycans from Gram-positive bacteria, bacterial lipoprotein, lipoteichoic acid, lipo-arabinomannan, zymosan, bacterial ciliated protein (flagellin), fusion protein from respiratory syncytial virus, non-methylated CpG nucleotides, double-stranded RNAs (dsRNAs) and single-stranded RNAs (ssRNAs), among others. If there is a TLR deficiency, these pathogens are not recognized, leading to various infectious immune diseases. Each TLR recognizes a different PAMP depending on where it is localized. TLR1, -2, -4, -5, and -6 are present on the cell surface, and thus detect extracellular PAMPs, whereas TLR3, -7, -8, and -9 are present in the cytosol and endosome membranes, and thus detect intracellular PAMPs. TLR3 recognizes viral dsRNA; TLR4 recognizes bacterial LPS; TLR5 recognizes flagella; TLR7/8 recognize ssRNA; TLR9 recognizes dsDNA; TLR3, -7, -8, and -9 recognize viral nucleic acid; and TLR2 and 4 recognize viral protein [12,17,18,19] (Table 1, Figure 1). 

Recent findings implicating TLRs in various diseases have spurred research on the role of TLRs. Studies on otitis media have suggested that altered expression of TLRs is involved in the etiology of OME. Accordingly, in the current review article, we systematically searched the literature using the keywords ‘otitis media’, ‘acute otitis media’, ‘otitis media with effusion’, ‘chronic otitis media without cholesteatoma’, ‘chronic otitis media with cholesteatoma’, and ‘TLR’ (Table 2). Literature databases were searched for studies published in English and were included if they: (1) were prospective and retrospective investigational studies; (2) included patients diagnosed with otitis media, AOM, OME, COM without cholesteatoma, or COM with cholesteatoma; and (3) included human patients or animal studies on TLR. Ultimately, we selected 26 articles satisfying search criteria for comprehensive review.

## 2. TLRs and Otitis Media 

### 2.1. TLR1

TLR1, also known as cluster of differentiation 281 (CD281), is a type of PRR in the innate immune system that recognizes PAMPs with a specificity for Gram-positive bacteria. TLR1 interacts with TLR2, forming a heterodimer that recognizes peptidoglycan and (triacyl) lipopeptides [46,47]. 

A study on TLR1 expression in relation to otitis media that compared the expression of TLR1 in the peripheral blood of recurrent or persistent AOM children with that of a normal pediatric control group showed that expression of TLR1 in monocytes was higher in the AOM group than in the normal control group [20]. In another study, in which OME children who had undergone ventilation tube insertion were classified into otitis-prone and non-otitis-prone groups, a comparison of TLR1 expression in middle ear effusion of both groups showed a trend toward higher expression of TLR1 mRNA in the otitis-prone group [21]. 

TLR1, which is expressed on the cell surface and reacts with bacterial-specific lipid proteins, is known to induce up-regulation of costimulatory molecules and pro-inflammatory cytokines through mitogen-activated protein kinase (MAPK) and NF-κB pathways [47]. The two studies mentioned above, taken together with these characteristics of TLR1, suggest that TLR1 is involved in recognizing pathogens in the initial infection state and inducing effective innate immune responses. However, it is thought that TLR1 does not likely contribute to recurrence or chronicization of otitis media.

### 2.2. TLR2

TLR2 is involved in the recognition of various pathogen structures, including lipoproteins, lipoteichoic acid, lipoarabinomannan, glycosylphosphatidylinositol anchors, phenol soluble modulin, zymosan, and glycolipids. In addition, some structural variants of LPS in *Leptospira interrogans* or *Porphyromonas gingivalis* are recognized by TLR2. Recognition of these variants by TLR2 requires cooperation with TLR1 or TLR6, and the resulting TLR2-TLR1 or TLR2-TLR6 heterodimers signal through the phosphoinositide 3-kinase (PI3K) pathway [22,23,47,48].

In animal experiments, TLR2 expression was shown to be higher in middle ear mucosa than in the nasopharynx mucosa or oral cavity mucosa of rats [24]. In another study, wild-type (WT) and TLR2−/− mice were inoculated in the middle ear with *Streptococcus pneumoniae* (Spn) serotype 19F, and the middle ear mucosa was collected and compared between the two groups. After Spn infection, TLR2 expression was significantly increased in the middle ear mucosa of WT mice. TLR2−/− mice were not affected in the early stages of infection, but in the late stage of infection, the absence of TLR2 resulted in a reduction in macrophage recruitment, poor Spn clearance and, ultimately, continued inflammation in the middle ear. On the basis of these results, the authors suggested that TLR2 signaling plays an important role in the effective clearance of bacteria and attenuation of inflammation in Spn-induced otitis media [22]. In another study using *S. pneumoniae*, infection was more severe on day 7 in TLR2−/− mice than in WT mice, blood bacterial titers were higher at 3 and 7 days, and more bacteria were detected in middle ear effusions, demonstrating that TLR2 plays an important role in the molecular pathogenesis and host response in otitis media [23]. Results similar to those with Spn were obtained in an otitis media model induced by non-typeable *Haemophilus influenzae* (NTHi), a common causative strain of AOM. In this study, bacterial clearance was impaired in TLR2−/− mice compared with that in the control group, indicating that TLR2 plays an important role in the regulation of infection in NTHi-induced otitis media [25]. 

In a study examining the expression of TLR in human AOM, OME, COM with cholesteatoma, and COM without cholesteatoma tissues, TLR expression was detected in tissues from each type of otitis media. In addition, expression of TLR2 was higher in the inflammatory mucosa with otitis media group compared with the normal control group [26]. A comparison of the expression of TLR2 in lymphocytes, monocytes, and granulocytes in blood from recurrent or persistent AOM pediatric patients showed that the expression of TLR2 was high in monocytes and low in lymphocytes. Collectively, these results suggest that increased TLR2 expression in peripheral blood monocytes is involved in the pathogenesis of AOM [20]. In a study that divided AOM children into otitis-prone and non-otitis prone groups, middle ear fluid was collected by tympanocentesis, and TICAM2 (Toll-like receptor adapter molecule 2) expression in both groups was compared using reverse transcription polymerase chain reaction (RT-PCR). This analysis revealed that TICAM2 expression was decreased by more than two-fold in the otitis-prone group [27]. A study that compared TLR2 levels between bacterial culture groups that tested positive and those that tested negative in bacterial culture assays showed a trend toward increased expression of TLR2 in the positive group compared with the negative culture group. However, quantitative PCR results comparing TLR2 levels for *S. pneumoniae*, NTHi and *M. catarrhalis* showed that TLR2 mRNA expression in the bacteria-positive group was more than 100 times higher than that in the bacteria-negative group [28]. In addition, TLR2 expression was confirmed in middle ear effusion of OME children who underwent ventilation tube insertion [29]. A study comparing the expression of TLR2 in middle ear effusion among otitis-prone and non-otitis-prone OME children who underwent ventilating tube insertion showed that expression of TLR2 mRNA was significantly lower in the otitis-prone group [21]. A comparison of the middle ear mucosa of the non-otitis media group, COM group, and chronic suppurative otitis-media (CSOM) group showed no difference in mRNA or protein levels of TLR2 between the non-otitis media and COM groups, but revealed a significant reduction in TLR2 expression in the CSOM group. This suggests that reduced TLR2 expression levels in the middle ear mucosa cause a persistent inflammatory response and weaken susceptibility to CSOM [30]. However, other studies have obtained different results, reporting that TLR2 is weakly expressed in normal middle ear samples and is up-regulated in COM and cholesteatoma compared with normal middle ear samples [31]. In addition, a comparison of TLR2 expression in normal canal skin, COM, and cholesteatoma showed higher TLR2 mRNA and protein levels in COM and cholesteatoma mucosa and granulation tissue than in normal external auditory canal skin [32,33].

A study of TLR2 polymorphisms reported that, among 381 AOM pediatric patients, TLR2 polymorphisms were associated with recurrent AOM [34]. However, a study of OME pediatric patients who underwent ventilation tube insertion reported no association with TLR2 mutations (Arg753Gln and Arg677Trp) [35]. 

### 2.3. TLR3

TLR3, which is mainly distributed in endosomes and recognizes double-stranded RNA (dsRNA) associated with viral infection, induces activation of interferon regulatory factor 3 (IRF3) and NF-κB. Unlike other TLRs, TLR3 uses Tir-domain-containing adaptor inducing interferon β (TRIF) as its sole adaptor. Upon ligand recognition, TLR3 induces activation of IRF3 to increase the production of type I interferons (IFNs), which signal other cells to increase their antiviral defenses. IRF3 ultimately induces the production of IFNs and may thus play a role in host defense against viruses [49,50].

In a study that measured TLR3 expression in the skin of patients with acquired cholesteatoma and those with a normal external auditory canal, TLR3 expression was weak in normal skin but was clearly detected in the skin of cholesteatoma patients, where it was mainly observed in the matrix (layer of keratinizing epithelium) and perimatrix (granulation tissue) [36]. 

### 2.4. TLR4

TLR4 is the first subtype identified in humans and the best-studied among the TLR family. TLR4 recognizes LPS, a cell wall component of Gram-negative bacteria, and endogenous ligands such as heat shock protein 60 (HSP60), fibronectin, and hyaluronic acid, which are often produced in response to stressful stimuli. TLR4 signaling in response to LPS begins with formation of protein complexes mediated by the extracellular leucine-rich repeat domain (LRR) and an intracellular toll/interleukin-1 receptor (TIR) domain in TLR4. LPS stimulation induces several proteins to interact with TLR4 to form complexes at the cell surface [18]. LPS recognition is initiated by binding to an LPS-binding protein (LBP). The resulting LPS-LBP complex transfers LPS to CD14, a glycosylphosphatidylinositol-anchored membrane protein that binds the LPS-LBP complex and facilitates the transfer of LPS to MD-2 protein, which is associated with the extracellular domain of TLR4. LPS binding thus promotes the dimerization of TLR4 and MD-2. TLR4 not only mediates responses to LPS, a major cellular component of Gram-negative bacteria, it is also involved in inflammatory responses to various other substances, such as ligands of harmful factors produced by Gram-positive bacteria and fusion protein (F protein) of respiratory syncytial virus [18,26,51]. In addition, individuals with a TLR4 D299G polymorphism are at increased risk of septic shock induced by Gram-negative bacteria infections.

Animal experiments showed that TLR4 expression is higher in the middle ear mucosa than in the nasopharynx mucosa or oral cavity mucosa of rats [24]. Another study that analyzed the expression of TLR4 in middle ear effusions and tissues after injection of NTHi—an important cause of middle ear infection—into the tympanic bulla of C3H/HeN mice, confirmed that TLR4 causes accumulation of polymorphonuclear cells in the early stage of inflammation and activates their functions to eliminate NTHi infections in the middle ear cavity [37]. It has also been reported that bacterial clearance is impaired in TLR4−/− mice compared with WT mice, indicating that TLR4 plays an important role in controlling middle ear inflammation in NTHi-induced otitis media [25]. 

Activated TLRs signal via two alternative intracellular signaling molecules with differing effects: MyD88, which primarily induces interleukin expression, and TRIF, which mediates expression of IFNs. Based on this, researchers used TLR4−/− and TRIF−/− mice to determine whether TRIF expression in the middle ear is altered by a TLR4 deficiency. These authors reported that TRIF expression in the absence of NTHi was significantly greater in both knockout strains than in uninfected WT mice, suggesting that activation of TLR3 or -4 by NTHi molecules triggers a series of signaling pathways through TRIF. In addition, TRIF mRNA expression was prominently reduced in TLR4-knockout mice compared with WT mice. On the basis of these results, the authors suggested that activation of TRIF/type I IFN responses is important in both the pathogenesis and resolution of NTHi-induced otitis media and that TLR4 may be involved in this process [38]. 

It has been shown that TLR4 is expressed in AOM, OME, COM with cholesteatoma, and COM without cholesteatoma, and is closely related to the etiology of otitis media [20]. A comparison of TLR4 expression in lymphocytes, monocytes, and granulocytes in peripheral blood from children with recurrent or persistent AOM showed that TLR4 expression is high in monocytes and low in lymphocytes. These results suggest that an increase in TLR4 in peripheral blood monocytes is involved in the pathogenesis of AOM [20]. An analysis of DNA samples from 348 children with a history of two or more AOM episodes and 463 healthy adults showed that the TLR4 299A/A genotype was associated with an otitis-prone condition [39]. In another study, middle ear fluid was collected by tympanocentesis from pediatric AOM patients, and the expression of TLR4 was analyzed after dividing samples into positive and negative bacterial culture groups based on the results of real-time PCR and bacterial culture tests of *S. pneumoniae*, NTHi and *M. catarrhalis*. The expression of TLR4 was significantly increased in the bacterial culture-positive group compared with the bacterial culture-negative group [28]. In addition, TLR4 expression was reported in middle ear fluid collected from pediatric OME patients who had undergone ventilation tube insertion [29]. Similarly, a study that compared the expression of TLR4 in middle ear fluid between otitis-prone and non-otitis-prone pediatric OME patients reported that expression of TLR4 mRNA was significantly lower in the otitis-prone group [21]. Moreover, a study investigating TLR2 and TLR4 expression and the presence of TLR4 mutants, Asp299Gly and Thr399Ile, reported lower expression of TLR4 compared with TLR2 and found no mutations in TLR4 [39].

A comparison of the middle ear mucosa of non-otitis media, COM, and CSOM groups showed that mRNA and protein levels of TLR4 were not different between non-otitis media and COM groups, but were significantly decreased in the CSOM group. These authors thus concluded that reduced TLR4 levels in the middle ear mucosa cause a persistent inflammatory response and weaken susceptibility to CSOM [30,40]. 

Studies have shown that TLR4 is not expressed in normal middle ear samples, but is readily detected in COM and cholesteatoma [26,31]. A comparison of TLR4 expression in normal canal skin, COM, and cholesteatoma showed that TLR4 mRNA and protein levels were increased in mucosa and granulation tissue of COM and cholesteatoma patients compared with those in normal external auditory canal skin [32].

A study that compared 53 selected SNPs in 35 genes in the blood of 624 children with otitis media and 778 patients without otitis media found that the single nucleotide polymorphism (SNP) rs5030717 in the TLR4 gene region was associated with the occurrence of otitis media. This study also showed that prevalence of this SNP was greater in children under 6 months of age and in children who underwent repeated insertion of tympanostomy tubes [41]. 

### 2.5. TLR5

Unlike TLR2, -4 and -6, which can mediate inflammatory responses to multiple microbial products, TLR5 and -9 recognize only one microbial structure. Of these, TLR5 specifically recognizes bacterial flagellin, which is the main structural protein in bacterial flagella; accordingly, only bacteria with flagella can activate TLR5. Flagellin does not contain any lipid or carbohydrate modifications, and TLR5 is expressed only on the basolateral side of intestinal epithelial cells. Thus, an inflammatory response to flagellin requires exposure to the basolateral side of the intestinal epithelium rather than the apical side. Upon activation of TLR5, flagellin stimulates the production of inflammatory mediators such as TNF-α. TLR5 is also very important in mucosal immunity because it can produce inflammatory cytokines by recognizing flagellin in intestinal epithelial cells and lung endothelial cells [52,53].

Another study investigated the expression of TLR5 in middle ear effusion collected from pediatric OME patients who had undergone ventilation tube insertion [29]. These authors reported that, among non-otitis media, COM, and chronic suppurative otitis-media (CSOM) groups, mRNA and protein levels of TLR5 were significantly reduced in the CSOM group, but were not different between non-otitis media and COM groups. Thus, reduced TLR5 expression levels in the middle ear mucosa cause a persistent inflammatory response and weaken susceptibility to CSOM [30]. 

### 2.6. TLR6

TLR6 is a PRR localized to the cell membrane that binds to multiple diacyl peptides derived from Gram-positive bacteria and mycoplasma, as well as several fungal cell wall saccharides. TLR6 functionally interacts with TLR2 to mediate cellular responses to Gram-positive bacteria, mycoplasma, fungi, some viruses, and even protozoa. After dimerization, TLR6-TLR2 heterodimeric complexes activate the NF-κB signaling pathway, leading to the production of pro-inflammatory cytokines and activation of the innate immune response [54,55].

Another study compared the expression of TLR6 in middle ear effusion after classifying pediatric OME patients who had undergone ventilation tube insertion into otitis-prone and non-otitis–prone groups. This study showed that the expression of TLR6 mRNA was significantly lower in the otitis-prone group than in the non-otitis–prone group, with similar results to those observed for TLR2 mRNA [21]. These results can be taken to mean that such interactions between TLR2 and TLR6 are involved in recognizing LPS in the pathophysiology of otitis media. 

### 2.7. TLR7

TLR7 triggers an immune response by recognizing imidazoquinolines, synthetic substances used to treat human papillomavirus infection. The structures of TLR7 and TLR8 are very similar, and both were assumed to recognize the nucleic acid-like structure of viruses. With the recent demonstration that TLR7 and TLR8 recognize the guanosine/uridine-rich ssRNA of human immunodeficiency virus (HIV) and influenza virus, it became possible to reliably classify the ligands of TLR7 and TLR8. However, although ssRNA is a structure that exists in hosts, TLR7 and TLR8 cannot recognize the host’s ssRNA because they are localized to the endosome. Experiments using plasmacytoid DCs (pDCs) from TLR7−/− mice, with or without chloroquine exposure, have shown that TLR7 recognizes ssRNA viruses (e.g., influenza virus, VSV, HIV) and that TLR7 and ssRNA react within the endosome [56]. An analysis of TLR7 polymorphisms in 381 children with AOM showed that TLR7 polymorphisms reduced the risk of rhinovirus-associated infection [34]. An investigation of TLR7 mRNA expression in adenoid tissues collected from 11 OME patients and 10 control individuals showed that TLR7 mRNA expression was increased in the OME group [42]. 

### 2.8. TLR8

TLR8 is an endosomal receptor that binds ssRNA and recognizes ssRNA viruses such as Influenza, Sendai, and Coxsackie B. TLR8 binding to viral RNA recruits MyD88 and leads to activation of the transcription factor NF-κB and induction of an antiviral response. TLR8 also recognizes ssRNAs of viruses such as HIV and HCV [57]. 

In a study investigating a role for TLR8 in the pathophysiology of otitis media, an analysis of TLR8 polymorphisms in 381 AOM children revealed that TLR8 polymorphisms were associated with an increased susceptibility to recurrent rhinovirus infections [34].

### 2.9. TLR9

TLR9 recognizes unmethylated CpG motifs, which are found much more abundantly in bacterial DNA than in vertebrate DNA and thus serve as PAMPs. Recognition of these unmethylated CpG motifs by TLR9 stimulates inflammatory responses. In vertebrates, the frequency of CpG motifs is very low, and because these motifs are methylated, they do not stimulate the immune system. When bacteria are engulfed by macrophages and DCs, they are degraded in the endosome, releasing their unmethylated CpG DNA. TLR9 is expressed within the endosome compartment, where it binds to this CpG motif-rich DNA and functions to warn the immune system of viral and bacterial infections [58]. Induction of TLR9 signaling in these cells triggers an inflammatory response, leading to the production of cytokines such as IFNs, IL-6, TNF, and IL-12 [49]. Notably, splenocyte proliferation and increased MHC II expression in B lymphocytes were not observed in TLR9−/− mice, despite CpG DNA stimulation. Moreover, production of TNF-α, IL-6, and IL-12 was decreased in macrophages of TLR9−/− mice, and expression of CD40, CD80, CD86 and MHC genes in DCs was not increased, again despite CpG DNA stimulation. These results indicate that CpG DNA stimulation does not induce an immune response in TLR9−/− mice, indicating that TLR9 plays an important role in the immune mechanisms induced by CpG DNA [43,59]. In a study comparing middle ear inflammation between WT and TLR9−/− mice (C57BL/6 background) following induction of otitis media by NTHi, TLR9−/− mice showed a sustained inflammatory response, but delayed bacterial clearance, in the middle ear [43]. TLR9 expression was also reported in middle ear effusion collected from pediatric OME patients who had undergone ventilation tube insertion [29]. Another study reported that TLR9 mRNA expression was lower in middle ear fluid collected from OME patients in an otitis-prone group than in non-otitis–prone patients [21,44]. Similarly, a comparison of TLR9 expression in the middle ear fluid of OME patients, with or without positive bacterial cultures, showed that expression of TLR9 mRNA was decreased in the positive bacterial culture group relative to the negative bacterial culture group [42]. In another study, 66 AOM children were divided into positive and negative bacterial culture groups based on results of bacterial culture tests of middle ear fluid collected through tympanocentesis, and TLR9 expression levels were assessed in the two groups. This analysis showed that TLR9 expression trended higher in the bacterial culture-positive group compared with the bacterial culture negative group [45]. Better evidence for such a TLR9 response was provided by comparing TLR9 expression levels of *S. pneumoniae*, NTHi and *M. catarrhalis* by quantitative real-time PCR, which showed a significant increase in the expression of TLR9 in bacteria-positive groups compared with bacteria-negative groups [30]. 

### 2.10. TLR10

Unlike other TLRs, TLR10 does not activate the immune system and has instead been shown to suppress inflammatory signaling in primary human cells. Thus, TLR10 is unique among TLR family members in having an anti-inflammatory function rather than a pro-inflammatory function. Activated TLR10 suppresses cytokine production compared with control cells and also exerts long-term effects on monocyte and B cell activation/differentiation by suppressing the transcription of activation markers. The mechanism of action of TLR10 is not yet clear, but activation of the receptor has been shown to suppress NF-κB, MAP kinase, and Akt signaling events stimulated by TLR and CD40 ligands [60,61]. TLR10 is mainly expressed in cells of the immune system, epithelial cells, and endothelial cells [34]. Given that immune cells in the middle ear cavity are abundantly generated during otitis media and epithelial cells and endothelial cells are present in the middle ear mucosa, TLR10, which is expressed in these cell types, is expected to play a role in otitis media, although no studies have yet directly investigated this.

## 3. Conclusions

To date, studies have mainly concentrated on the pathophysiology, complications and prognosis of otitis media under conditions of TLR overexpression and deficiency. Research in this area has sought to identify compounds that can control TLR expression or specific TLR substance related to otitis media, microorganisms, and cytokines which can lead to improved prognosis, fewer complications and early treatment of AOM, OME, and COM with or without cholesteatoma. 

Abnormal TLR signaling is known to be associated with diseases such as sepsis, immunodeficiency, atherosclerosis, asthma, and auto-immune diseases. As we show in this review, TLRs are also expressed in AOM, OME, COM with cholesteatoma, and COM without cholesteatoma, suggesting their involvement in immune responses in the middle ear in otitis media. TLRs show little or no expression in the normal middle ear mucosa, but their expression increases or decreases depending on the presence or absence of bacteria in the middle ear cavity, recurrence of otitis media, the types of tissues that mainly exhibit inflammatory reactions, and with repeated surgeries following the recurrence of otitis media. Thus, TLR is very closely related to immune responses of the middle ear in otitis media such that inappropriate expression of TLRs in cells related to the immune response in middle ear tissue or blood, or defects in the corresponding response, may promote the occurrence, recurrence or chronicization of otitis media, or exacerbate its consequences and lead to complications. Therefore, TLRs are key factors in the innate immune response that are likely very important in the pathogenesis of otitis media.

## Figures and Tables

**Table 1:** TLR recognition of microbial components.

TLR	Species	Components
TLR1	Bacteria and mycobacteria	Triacyl lipopeptides
	*Neisseria meningitidis*	Soluble factors released by live bacteria
TLR2	Mycoplasma	Diacyl lipopeptides
	Bacteria and mycobacteria	Triacyl lipopeptides
	Group B Streptococcus	LTA
	Gram-positive bacteria	PG
	Neisseria	Porins
	*Neisseria meningitidis*	Soluble factors released by live bacteria
	Mycobacteria	Lipoarabinomannan
	*Saccharomyces cerevisiae*	Zymosan
	Yeasts	Zymosan
	*Candida albicans*	Phospholipomannan
	*Cryptococcus neoformans*	Glucuronoxylomannan
	Trypanosoma	tGPI-mutin
	*Trypanosoma cruzi*	Glycosylphosphatidylinositol anchors
	Measles virus	Hemagglutinin protein
	HCMV, HSV1	ND (Not determined)
	Several bacterial species	Lipoprotein
	*Staphylococcus aureus*	Lipoteichoic acid
		Phosphatidylinositol dimannoside
		Soluble phenol modulin
	*Leptospira interrogans*	Endotoxin (LPS)
	*Porphyromonas gingivalis*	Endotoxin (LPS)
	*Mycoplasma fermentans*	Macrophage activating lipopeptide-2(MALP-2)
TLR3	Viruses	dsRNA
	Gram-positive bacteria	Peptidoglycan
	*Staphylococcus aureus*	Soluble phenol modulin
	Yeasts	Zymosan
	*Mycoplasma fermentans*	Macrophage activating lipopeptide-2(MALP-2)
TLR4	Gram-negative bacteria	LPS
	*Candida albicans*	Mannan
	Trypanosoma	Glycoinositolphospholipids
	RSV, MMTV	Envelope proteins
	Gram positive bacteria	Peptidoglycan
	*Staphylococcus aureus*	Soluble phenol modulin
	Yeasts	Zymosan
	*Mycoplasma fermentans*	Macrophage activating lipopeptide-2 (MALP-2)
	Plants	Taxol
	Heat-shock protein 60,70	-
	Fibrinogen	-
TLR5	Flagellated bacteria	Flagellin
	Gram-positive bacteria	Peptidoglycan
	*Staphylococcus aureus*	Soluble phenol modulin
	Yeasts	Zymosan
	*Mycoplasma fermentans*	Macrophage activating lipopeptide-2 (MALP-2)
TLR6	Mycoplasma	Diacyl lipopeptides
	Group B Streptococcus	LTA
	*Saccharomyces cerevisiae*	Zymosan
	Yeasts	Zymosan
	Gram-positive bacteria	Peptidoglycan
	*Staphylococcus aureus*	Soluble phenol modulin
	*Mycoplasma fermentans*	Macrophage activating lipopeptide-2 (MALP-2)
TLR7	RNA viruses	ssRNA
	Chemical compounds	Imidazoquinoline antiviral compounds (imiquimod and R-848)
TLR8	RNA viruses	ssRNA
TLR9	Bacteria and mycobacteria	CpG-DNA
	Plasmodium	Hemozoin
	Viruses	DNA
TLR10	?	?

TLR, Toll-like receptor; LTA, lipoteichoic acid; PG, Peptidoglycan; DNA, Deoxyribonucleic Acid; RNA, Ribonucleic Acid; ?, has not been extensively studied.

**Table 2:** Studies assessing the association between otitis media and Toll-like receptors.

Author [Reference]	Associated Diseases	Study Design	Species and/or Sample	Detection Method	Target Gene(s) or Pathway(s) Associated with TLRs	Results/Conclusion
Trzpis K et al. [20]	rAOM	Prospective study	Human: Peripheral blood	flow cytometric analysis	TLR1, TLR2, TLR4	Expression of all examined TLRs on monocytes was significantly higher in the AOM group. Peripheral blood monocytes are characterized by increased expression of TLRs in the course of recurrent AOM.
Lee HY et al. [21]	OME	Prospective study	Human: Middle ear fluids	RT- PCR	TLR1, TLR2, TLR4, TLR5, TLR6, TLR9	Expression levels of TLR-2, -4, -6, and -9 mRNA were significantly lower in the otitis-prone than in the non-otitis-prone group. Decreased expression of TLRs may be associated with increased susceptibility to OME.
Huang Y et al. [22]	AOM	Animal study	Mice	qRT-PCR, immunofluorescence	TLR2	TLR2 expression in ME mucosa was markedly enhanced following infection with *Streptococcus pneumoniae* in wild-type mice. TLR2 signaling is critical for bacterial clearance and timely resolution of inflammation in AOM induced by *Streptococcus pneumoniae*.
Han F et al. [23]	AOM	Animal study	Mice	RT-PCR, Hematoxylin/eosin-stain	TLR2	The histological pathology was characterized by effusion and tissue damage in the middle ear and, in TLR2−/− mice, the outcome of infection became more severe at 7 days. At both 3 and 7 days postchallenge, TLR2−/− mice had higher blood bacterial titers than WT mice. TLR2 is important in the molecular pathogenesis and host response to AOM.
Song JJ et al. [24]	Normal tubotympanum	Animal study	Mice	RT-PCR, Western blot analysis	TLR2, TLR4	Expression of TLR2 and TLR4 in the middle ear was increased more than in other anatomical areas. Differential expression of subtypes of the TLR in the normal physiology of the tubotympanum and upper aerodigestive tract also suggests that they may play a role in the pathophysiology of OM.
Leichtle A at al. [25]	OM	Animal study	Mice	DNA Microarray, Immunohistochemistry, Quantitative PCR	TLR2, TLR4	TLR2−/− and TLR4−/− mice exhibited more profound, persistent inflammation with impaired bacterial clearance compared to controls. TLR4 signaling appears to induce TLR2 expression, and TLR2 activation is critical for bacterial clearance and timely resolution of OM.
Jesic S et al. [26]	Chole OM	Prospective study	Human: Middle ear mucosa	Semiquantitative immunohistochemical methods	TLR 2, TLR 4	Stronger expression of TLR2 and -4 was found in inflamed mucosa than in controls in children and adults, in cholesteatoma perimatrix compared to tubotympanic lesions in children and adults. TLR2 and TLR4 mediate inflammation in cholesteatoma and mucosal lesions of tubotympanic otitis in children and adults.
Kaur R et al. [27]	AOM	Prospective, longitudinal study	Human	RT-PCR	TLR2	Expression of all examined TLRs on monocytes was significantly higher in the AOM group. Peripheral blood monocytes are characterized by increased expression of TLRs in the course of recurrent AOM.
Kaur R et al. [28]	OME	Prospective study	Human: MEF	RT-PCR	TLR2, TLR4, TLR9	Expression levels of TLR2, -4, -6, and -9 mRNA were significantly lower in the otitis-prone than in the non-otitis-prone group. Decreased expression of TLRs may be associated with increased susceptibility to OME.
Lee SY et al. [29]	OME	Prospective study	Human: MEF	RT-PCR	TLR2, TLR4, TLR5, TLR9	All effusion fluid samples collected from patients with OME showed expression of TLR2, -4, -5, -9 mRNA by PCR. Exudates of OME patients show TLR expression levels that are related to the innate immune response regardless of the characteristics of effusion fluid, presence of bacteria in exudates, or frequency of ventilation tube insertion.
Si Y et al. [30]	COM, CSOM	Prospective study	Human: ME mucosa	RT-PCR, Western blot	TLR2, TLR4, TLR5, TLR9	mRNA and protein levels of TLR2, -4, and -5 exhibited no difference between the non-OM and COM groups but were significantly lower in the CSOM group. Reduced TLR levels in the middle-ear mucosa might cause weak host response to bacteria, persistent inflammation and susceptibility to CSOM.
Hirai H et al. [31]	COM, chole OM	Prospective study	Human:ME tissue	Immunohistochemistry	TLR2, TLR4	Both TLR2 and -4 were markedly expressed in COM and chole OM. There was a significant difference between COM and normal controls in the expression of both TLRs. TLRs may play a principal role in human COM and chole OM.
Si Y et al. [32]	CSOM	Prospective study	Human: normal canal skin, mucosa and granulation tissue	RT- PCR, Western blot, Immunohistochemistry	TLR2, TLR4	Both mRNA and protein levels of TLR2 and -4 in mucosa of CSOM and chole OM were higher than those in normal canal skin, but lower than those in chole OM epithelium. There was no significant difference in mucosa of the two OM groups. Differential expression of TLR2 and -4 in mucosa suggests that they may play a different role in the pathophysiology of COM and chole OM.
Komori M et al. [33]	OME	Human and animal study	Human and animal (rat and mouse) specimens	Quantitative PCR, Immunohistochemistry, Western blot	TLR2	Expression of TLR2 was activated in ME epithelial cells through the NF-κB cytokine signaling pathway, while the I kappa B alpha mutant (I*κ*B*α*M), a dominant negative inhibitor of NF-κB, abrogated the expression of TLR2 induced by PGPS.
Toivonen L et al. [34]	AOM	Prospective cohort study	Human	PCR	TLR2 Arg753Gln, TLR3 Leu412Phe, TLR4 Asp299Gly, TLR7 Gln11Leu and TLR8 Leu651Leu.	TLR2 polymorphisms were associated with recurrent AOM. TLR7 polymorphisms were associated with a decreased risk of rhinovirus-associated AOM. Genetic polymorphisms in TLRs promote susceptibility to or protection against respiratory infections.
Lee YC et al. [35]	OME	Prospective study	Human: MEF	RT-PCR	TLR2, TLR4	TLR2 and -4 were expressed in the MEF and the expression of TLR2 was higher than that of TLR4. TLR2 and -4 were expressed in all MEF samples of OME, but the mutations of TLR 2 and 4 were not detected.
Szczepański M et al. [36]	Chole OM	Prospective study	Human: cholesteatoma and normal external auditory canal skin	Immunohistochemistry	TLR2, TLR3, TLR4	All TLRs tested were demonstrated in matrix (layer of keratinizing epithelium) and perimatrix (granulation tissue) of this inflammatory tumor. Weak expression of these receptors on normal skin may also suggest the important role of TLRs in the etiopathogenesis of cholesteatoma.
Hirano T et al. [37]	AOM	Animal study	C3H/HeJ mice: ME mucosa	H&E staining, Confocal laser scanning microscopy	TLR4	In WT mice, PMNs that had infiltrated the ME mucosa showed strong immunostaining of both TLR2 and -4 24 h after NTHi injection. In TLR4 deficient mice, PMNs showed hardly any staining of TLR2 and -4. TLR 4 plays a part in the early accumulation and functional promotion of PMNs in the ME for eradicating NTHi infection.
Leichtle A at al. [38]	OM	Animal study	Mice	Quantitative PCR, DNA Microarray	TLR adaptor TRIF	Expression of TRIF mRNA was only modestly enhanced during OM. TRIF-deficient mice showed reduced but more persistent mucosal hyperplasia and less leukocyte infiltration into the ME in response to NTHi infection than did WT animals. Activation of TRIF/type I IFN responses is important in both the pathogenesis and resolution of NTHi-induced OM.
Emonts M et al. [39]	AOM	Randomized, controlled trial	Human DNA	PCR	TLR2, TLR4	TLR4 299 A/A genotype was associated with an otitis-prone condition. Variation in innate immunoresponse genes contributes to an otitis-prone condition.
Tuoheti A et al. [40]	CSOM, COM and non-OM	Prospective study	Human and C57BL/6 mice	qRT-PCR, Western blot	TLR2,TLR4, TLR5, TLR9	TLR4, instead of other TLRs, showed low expression in the CSOM group compared to the COM and non-COM groups. TLR4 deficiency promoted chronic inflammation in LPS-induced acute otitis media mice models. Knock-down of Nrf2 reversed chronic inflammation to attenuate CSOM by up-regulating TLR4.
Hafrén L et al. [41]	rAOM or OME	Cohort study	Human: DNA was extracted from peripheral blood	SNP	TLR4 gene	SNP rs5030717 in the TLR4 gene region showed significant association to OM. The TLR4 gene locus, regulating the innate immune response, influences the genetic predisposition to childhood OM.
Granath A et al. [42]	OME	Controlled, prospective study	Human: adenoid tissue	qRT-PCR, Immunohistochemistry	TLR7	mRNA levels for TLR7 were increased among children with a history of OME.
Leichtle A et al. [43]	Otitis media	Animal study	C57Bl/6:CB F1 hybrid mice	qRT-PCR, Immunohistochemistry	TLR9	TLR9 deletion significantly prolonged the inflammatory response induced by NTHi in the ME and delayed bacterial clearance. The results suggest that DNA sensing via TLR9 plays a role in OM pathogenesis and recovery.
Kim MG et al. [44]	OME	Prospective study	Human: MEF	RT-PCR	TLR9	The levels of TLR9 mRNAs were significantly lower in the otitis-prone than in the non-otitis-prone group. Decreased expression of TLRs may be associated with increased susceptibility to OME.
Lee HY et al. [45]	OME	Prospective study	Human: MEF	RT-PCR	TLR9	Down-regulation of TLR9 was observed in the culture-positive group. The expression of TLR9 significantly decreased in OME with confirmed bacterial pathogens.

TLR, Toll-like receptor; rAOM, recurrent acute otitis media; AOM, acute otitis media; OME, otitis media with effusion; RT-PCR, reverse transcription-polymerase chain reaction; qRT-PCR, quantitative reverse transcription-polymerase chain reaction; ME, middle ear; WT, wild-type; OM, otitis media; Chole OM, chronic otitis media with cholesteatoma; MEF, middle ear fluid; COM, chronic otitis media; CSOM, chronic suppurative otitis media; PGPS, peptidoglycan-polysaccharide; PMNs, polymorphonuclear cells; NTHi, Nontypeable Haemophilus influenzae; TRIF, TIR-domain-containing adapter-inducing interferon-β; IFN, interferon; LPS, lipopolysaccharide; SNP, single nucleotide polymorphism.
